# Can Herbivore Feeding Preferences Reinforce the Female‐Biased Sex Ratio in an Alpine Willow?

**DOI:** 10.1002/ece3.73816

**Published:** 2026-06-08

**Authors:** I. C. Barrio, C. G. Bueno, D. S. Hik

**Affiliations:** ^1^ Department of Biological Sciences University of Alberta Edmonton Alberta Canada

**Keywords:** dioecious plants, Lagomorpha, Lymantriidae, *Salix arctica*, sex‐biased herbivory, tundra

## Abstract

Sex‐biased herbivory is a potential mechanism for maintaining female‐biased sex ratios in Arctic and alpine willows, which are consumed by taxonomically diverse herbivores. Differences in feeding preferences of these herbivores may determine synergistic or antagonistic pressures on populations of their shared food plants. We investigated the foraging preferences of two alpine herbivores, a caterpillar and a small mammal, both feeding on the widespread, dioecious tundra shrub *Salix arctica* , at different times of the growing season. The caterpillars of the Arctic moth *Gynaephora groenlandica beringiana* are active in the early summer, while collared pikas *Ochotona collaris* are most active later in the summer. We used cafeteria‐type experiments to evaluate preferences for male and female leaves of *S. arctica* and found that the two herbivores showed different patterns in their feeding preferences. Caterpillars preferred male plants in early summer, feeding on male leaves nearly twice as much as female leaves, while collared pikas showed no preference between undamaged male and female *S. arctica* leaves later in the season. Sex‐biased herbivory by sequential, co‐occurring herbivores may have consequences for the population dynamics of dioecious plants. The cumulative effects of these different herbivores on plant population dynamics should be considered in highly seasonal environments, particularly if the relative balance of caterpillar and pika herbivory changes in the future.

## Graphical abstract

Sex‐biased herbivory can vary among co‐occurring herbivores and across the season. Sequential herbivory by species with different feeding preferences may influence the population dynamics of dioecious plants, particularly if early‐season herbivory alters plant traits that affect subsequent foraging decisions.

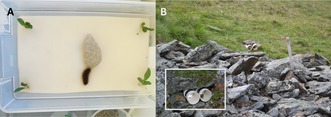

## 1 Introduction

Dioecy is a common strategy in alpine and arctic plants, particularly among early‐flowering species, with approximately 20% of species having male and female individuals (Molau 1993). Differential consumption of male and female plants by herbivores, or sex‐biased herbivory, has been suggested as a potential evolutionary force in the development and maintenance of dioecy in natural communities (Ashman 2002). Earlier studies suggested that male‐biased herbivory is the more common pattern in many ecosystems (Cornelissen and Stiling 2005), although the generality of these results has been questioned (Avila‐Sakar and Romanow 2012; Sargent and McKeough 2022), and preferences seem to differ between herbivores (Boecklen et al. 1994).

Willow populations in northern areas generally show female‐biased sex ratios (Myers‐Smith and Hik 2012). Willows provide food for many arctic herbivores, and sex‐biased herbivory may contribute to maintaining the biased sex ratios in northern willows in some circumstances (Elmqvist et al. 1988; Danell et al. 1991). Evidence for male‐biased herbivory in northern latitudes comes mainly from boreal forests and has been restricted to a few herbivore species ( *Microtus agrestis* and *Clethrionomys glareolus* ; Danell et al. 1985, 1991; Elmqvist et al. 1988; *Lepus timidus* ; Hjältén 1992), including invertebrates (Ågren 1987). In tundra, evidence for sex‐biased herbivory is more limited. Two studies on tundra willows reported no preferences for male or female plants by lemmings ( *Dicrostonyx groenlandicus* and *Lemmus lemmus* ) on *Salix lanata* (Predavec and Danell 2001), nor by muskox ( *Ovibos moschatus* ) or invertebrate herbivores on *Salix arctica* (Tolvanen et al. 2002). However, a study investigating gall‐forming mites reported higher rates of infestation on female plants of *S. arctica* (Mosbacher et al. 2013).

Since northern willows provide food for a range of herbivores, herbivore feeding preferences may impose competing selective pressures. In turn, feeding preferences of herbivores can be particularly important in highly seasonal environments, like tundra, where early season herbivores can affect food choices of other herbivores later in the season (Danell and Huss‐Danell 1985; Barrio, Hik, et al. 2013), and plant quality strongly changes over the growing season (Petit Bon et al. 2022). However, studies investigating the effects of multiple, sequential herbivores on plants rarely take into account sex differences of dioecious plants. We investigated the feeding preferences of two co‐occurring alpine herbivores that feed on a widespread, dioecious tundra willow, *S. arctica* , at different times of the growing season. We used an early‐season invertebrate herbivore, the Arctic moth *Gynaephora groenlandica* , and a late‐season vertebrate herbivore, the collared pika *Ochotona collaris* . This plant‐herbivore system provides a good model to investigate the combined effects of different herbivores because the two herbivores have a high degree of overlap in spatial and temporal (seasonal) resource‐use, and the feeding activity of caterpillars is known to affect the foraging decisions of collared pikas (Barrio, Hik, et al. 2013). Further, Arctic moth caterpillars can affect the reproductive success and vegetative growth of *S. arctica* (Mølgaard and Morewood 1996; Barrio et al. 2016) and *S. arctica* is a preferred forage species of pikas (Hudson et al. 2008).

Given the physiological differences between sexes in *S. arctica* (Dawson and Bliss 1989; Jones et al. 1999), we hypothesize that male and female plants would represent different food quality for the herbivores. Irrespective of these potential differences, we predicted no sex‐biased preference for caterpillars, as caterpillars of the Arctic moth are generalist herbivores at our site (Barrio et al. 2015), and previous studies have shown no differences in the intensity of invertebrate herbivory on male and female *S. arctica* (Tolvanen et al. 2002). Pikas are also generalist herbivores, but their foraging choices are known to be affected by intraspecific variations in forage quality (Koh and Hik 2007; Morrison and Hik 2008). We therefore predicted that, when presented with undamaged leaves of *S. arctica* , pikas would select male willows, which seem to be more palatable to vertebrate herbivores like voles (Danell et al. 1985) and hares (Hjältén 1992). Evidence for sex‐biased herbivory toward higher consumption of male plants by both or even just one of the herbivores could reinforce the observed patterns of female‐biased sex ratios in alpine willow populations through differential adult mortality of male plants (Elmqvist et al. 1988; Predavec and Danell 2001), or through the existence of mechanisms during pollination or seed development that influence the number of individuals of each sex that establish (Myers‐Smith and Hik 2012).

## 2 Materials and Methods

### 2.1 Study Site and Species

The study was conducted in an alpine valley of the Ruby Range, SW Yukon, Canada (61°12′ N, 138°16′ W; elevation 1500–2000 m a.s.l.) in summer 2013. Alpine meadows and tundra vegetation are interspersed with boulderfields. Plant communities are dominated by *Salix spp*, *Cassiope tetragona* , *Dryas octopetala* and several graminoids (*Carex* spp.). We examined forage preferences for two common herbivores at this site, the Arctic moth, *G. groenlandica* , and the collared pika, *O. collaris* , which feed on the Arctic willow, *S. arctica* Pall., a dioecious prostrate dwarf shrub, with a wide geographical distribution and ecological amplitude. As in other parts of the Arctic (Dawson and Bliss 1989), in our study area *S. arctica* has a female‐biased sex ratio (J. Cameron, personal communication).

The Arctic moth has been identified as one of the main invertebrate herbivores in the Arctic tundra (Mølgaard and Morewood 1996). Throughout its range, caterpillars of the Arctic moth feed mostly on *S. arctica* ; the subspecies present at our site, *G. g. beringiana* (Barrio, Schmidt, et al. 2013) feeds heavily on *S. arctica* but has a broader diet spectrum (Barrio et al. 2015). The extended life cycle of this species (up to 7 years; Morewood and Ring 1998) is mostly determined by low temperatures and biotic factors, such as the presence of parasitoids and the quality of their host plants (Kukal and Kevan 1987). Caterpillars are active for a few weeks after snowmelt, after which they spin hibernacula to avoid the seasonal peak in parasitoid populations and become dormant until the next spring (Kukal and Kevan 1987).

Collared pikas are also abundant in the area and have significant impacts on vegetation productivity and composition (McIntire and Hik 2002, 2005). Pikas inhabit boulderfields and use the adjacent vegetation patches to forage, where they overlap with Arctic moth caterpillars (Barrio, Hik, et al. 2013). Pikas are generalist herbivores that feed on a variety of plants (Dearing 1996). When caching foods for winter during the plant biomass peak in late summer, pikas are selective in their foraging choices (Hudson et al. 2008; Barrio, Hik, et al. 2013) and intraspecific variation in forage quality has been shown to influence their foraging decisions (Morrison and Hik 2008).

### 2.2 Feeding Experiments

Forage selection by herbivores was assessed using cafeteria‐type experiments, where herbivores were presented with samples of male and female plants from which they could choose to eat. Leaf samples of *S. arctica* were collected immediately before the experiments from plants of known sex, as determined by the presence of male or female flowers. Eight male and eight female plants were marked for leaf collection for the caterpillar and pika feeding experiments. All plants were similar in size and age and located in a south facing alpine meadow at 1645 m a.s.l. Plants were not assessed for previous herbivory, but we ensured that sampled leaves had no signs of previous herbivory (i.e., undamaged leaves). On average, leaf samples consisted of groups of four undamaged leaves (range 1–8) and ~1 cm of woody stem, and were collected from random parts of the plant, ensuring that samples collected from the same individual would be at least 20 cm apart. A maximum of three leaf samples were collected from each shrub on a single day, for a maximum of three consecutive days, to minimize the potential impact of leaf collection on the plants.

**FIGURE 1:**
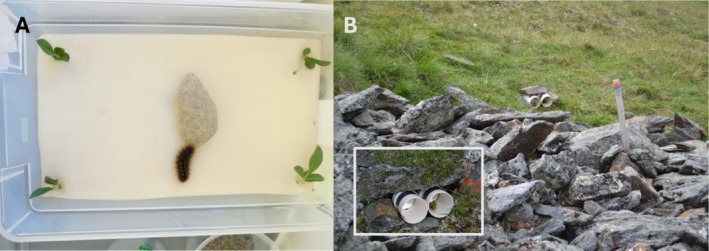
Forage selection of male or female *Salix arctica* plants by herbivores was assessed using cafeteria‐type experiments. (A) Cafeteria experiments with caterpillars were conducted indoors; caterpillars were presented with male and female leaves placed in the corners of a rectangular plastic container. (B) Cafeteria experiments for pikas were conducted outdoors and consisted of two PVC tubes (inset) from which pikas could select male or female leaves, while having access to other food sources.

For caterpillars, cafeteria experiments were conducted in an unheated shelter at ambient temperature. 36 caterpillars were collected in mid‐June at the study site and reared indoors in plastic pots (500 mL). To avoid affecting feeding preferences of caterpillars due to prior experience (Pérez‐Harguindeguy et al. 2003), all caterpillars were fed a varied diet of common tundra plants known to be part of their diet until 24 h before the experiment (Barrio et al. 2015). Cafeterias were set up with four leaf samples, placed in 1.5 mL water‐filled vials in the corners of a rectangular (34 × 20 × 10 cm) plastic container (MacLean and Jensen 1985; see Barrio et al. (2015) for detailed procedures; Figure 1A). Dry leaves were removed from the leaf samples, ensuring that caterpillars would have access to fresh leaves only. One caterpillar was placed in each cafeteria, where it was presented with four leaf samples belonging to four different plants (two male and two female). The position of the plant samples (male vs. female) was randomized. Each caterpillar was used only once in the experiment, and only caterpillars that ate during the cafeteria trials were included in the analyses (12 caterpillars). Trials started at the same time of day and lasted for 24 h, with 4 cafeterias running simultaneously, and were conducted between July 1–15, 2013. The proportion of herbivory on each plant sample was calculated as the number of leaves with signs of caterpillar herbivory out of the initial number of leaves; thus, for each caterpillar we had four observations (one per plant sample).

Cafeteria experiments for pikas were conducted in the field later in the season, between August 1–10, 2013, adjacent to boulderfields where pikas were actively collecting plants for their winter haypiles (Morrison et al. 2004, 2009). Over three consecutive days, 30 pikas were presented with two PVC tubes located at least 2 m from their haypiles (Figure 1B); similar to the caterpillar trials, each pika was only used once in each trial, but each pika trial lasted 3 days. Each tube contained five undamaged leaves of *S. arctica* of either one male or one female plant, from the same plant individuals as used for the caterpillar experiment. Leaves were replaced every 24 h to avoid excessive desiccation, and fresh leaves of different plant individuals were placed in the tubes. Thus, over the 3 days, each pika was exposed to six plant individuals (three males, three females). Only pikas that took at least one leaf from the tubes during the 3‐day trial were included in further analyses (28 pikas). The number of leaves removed by pikas from each tube was recorded each day. For analyses, trials for the three consecutive days for each pika were pooled and herbivory on male and female plants was calculated as the total number of leaves removed from the tubes out of 15 male and 15 female leaves.

### 2.3 Statistical Analyses

Data were analyzed using Generalized Linear Mixed Effects models (GLMM) with a binomial error structure for proportional data (Zuur et al. 2009), where plant sex was included as a fixed factor. The response variables for the caterpillar and pika GLMMs were specified as the number of affected leaves (either damaged by caterpillars or removed by pikas) and the number of unaffected leaves. Caterpillar or pika identity was included as a random factor to account for the nested data structure (four observations per caterpillar, i.e., one per plant sample in the cafeteria trials, and two observations per pika, i.e., one observation for male leaves and one for female leaves pooled over the 3 days of the cafeteria trials). All analyses were conducted in R 4.2.1 (R Development Core Team 2023), using the package *lme4* for building GLMMs. Visual inspection of model residuals confirmed that all modeling assumptions were met (Zuur et al. 2009; Figure S1). Data are reported as means and standard errors.

## 3 Results

**FIGURE 2:**
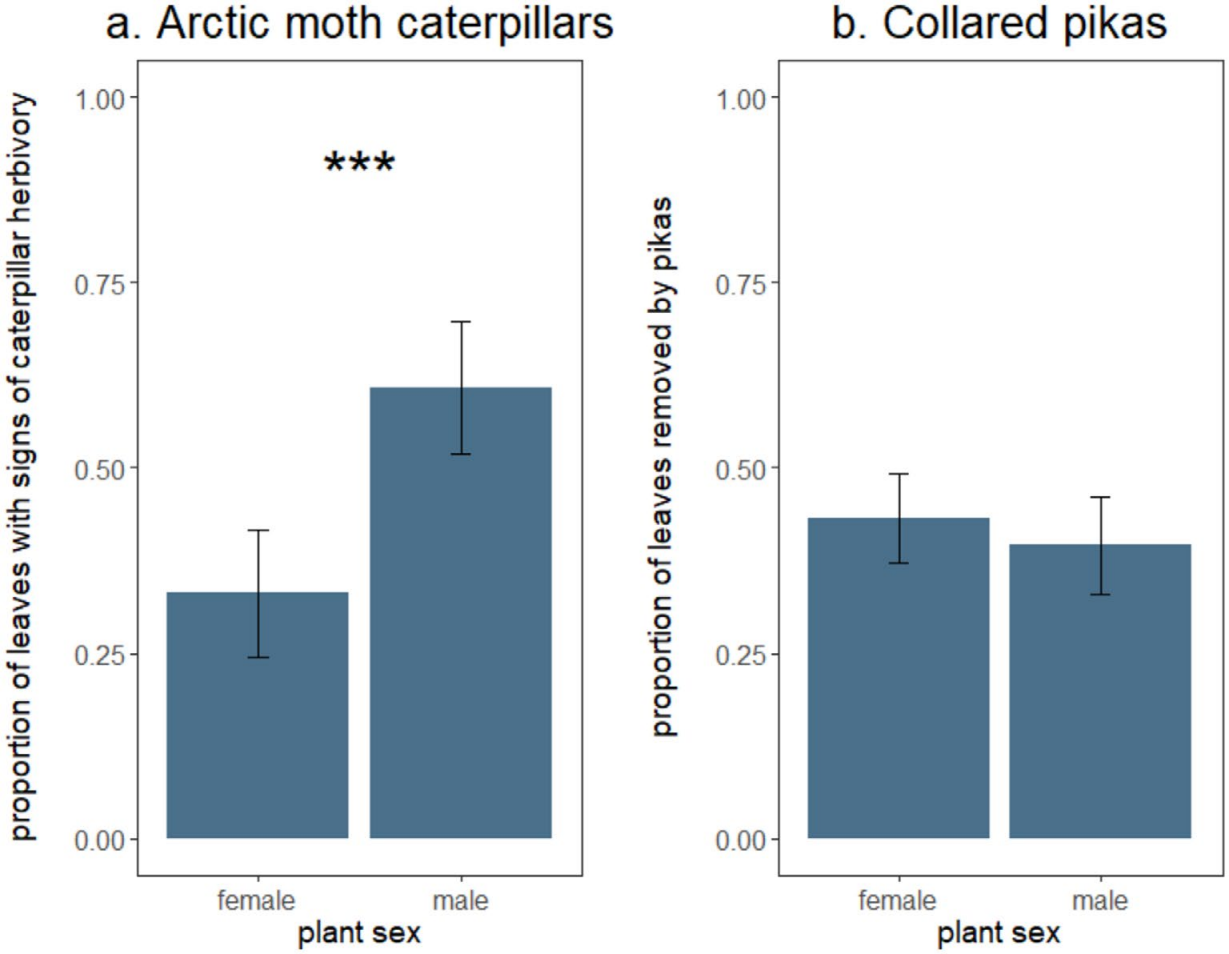
Plant sex selection of *Salix arctica* by two alpine herbivores: (a) Arctic moth caterpillars, *Gynaephora groenlandica* , and (b) collared pikas, *Ochotona collaris* . Selection was assessed in cafeteria‐type experiments; in the case of caterpillars, herbivory is expressed as the proportion of leaves with signs of caterpillar herbivory, and in the case of pikas, as the proportion of leaves removed from tubes containing leaves of each sex. Bars show means and standard errors (caterpillars: *N* = 24; pikas: *N* = 28). Asterisks indicate significant differences (***< 0.001) between male and female plants, as assessed with Generalized Linear Mixed models.

Overall, Arctic moth caterpillars fed on 46.9% ± 10.0% and pikas removed 41.3% ± 5.8% of the leaves presented to them in the cafeteria trials. Arctic moth caterpillars showed a preference to forage on male *S. arctica* leaves (GLMM, *z* value = 4.159, *p* < 0.001; Figure 2a), feeding on male leaves nearly twice as much as female leaves (proportion of herbivory on males: 60.8% ± 8.0%, *n* = 24; females: 33.1% ± 8.1%, *n* = 24). In contrast, pikas showed no difference in their preference between undamaged leaves of male and female willows (GLMM, *z* value = −1.322, *p* = 0.186; Figure 2b), although they removed a slightly higher proportion of female *S. arctica* leaves from cafeterias. On average, 43.1% ± 6.0% leaves were removed from tubes containing female leaves compared to 39.5% ± 6.7% from tubes containing male leaves.

## 4 Discussion

Our results show different preferences of alpine herbivores that use male and female *S. arctica* at different times of the growing season. The earlier herbivore showed a clear preference for male leaves, while the later herbivore showed no preference for undamaged leaves of male or female plants. The reasons for these differences could be related to differences in what the herbivores are using the plants for (e.g., caterpillars are directly consuming the leaves, while pikas are primarily collecting plants for overwinter storage), so their preferences could be driven by different considerations. In any case, a sex preference in plant use even by a single herbivore could help maintain the observed female‐biased sex ratio in *S. arctica* .

Male‐biased herbivory on dioecious plants has been widely reported in the literature and was assumed to be the most common pattern (Cornelissen and Stiling 2005). However, the generality of this statement has been questioned in recent syntheses (Avila‐Sakar and Romanow 2012; Sargent and McKeough 2022). Different herbivores have different patterns of plant use (Boecklen et al. 1994), and their foraging preferences may be more related to spatial and temporal variation in plant populations than to plant sex (Leal et al. 2023). Preferences by herbivores can also vary depending on food availability and habitat productivity. For example, field voles *M. agrestis* showed a stronger preference for male willows in areas with low herbivore pressure and moderate productivity, whereas no sex‐biased herbivory was detected in more productive habitats (Danell et al. 1991).

In our study, we minimized these sources of variability by conducting cafeteria trials under standardized conditions and using the same population of origin of plant samples, so differences in plant sex selection by the two herbivores might arise from actual differences in their feeding preferences. In addition, our experimental design attempted to incorporate phenological differences in plant quality that sequential herbivores face under natural conditions by conducting trials at times of the growing season when each herbivore is foraging most actively. Cafeteria trials for caterpillars were conducted early in the growing season, when caterpillars are active (Barrio, Hik, et al. 2013), while trials for pikas were conducted later, during the period of most active forage collection for overwinter storage (Morrison et al. 2004, 2009). The Arctic willow accumulates secondary metabolites over the growing season (Kukal and Dawson 1989), and leaf nitrogen declines over the season at different rates for male and female plants (Dawson and Bliss 1993). These phenological changes in plant quality may have influenced the preferences of our herbivores. Seasonal changes in feeding preferences for a single herbivore have been highlighted by recent studies (Valdés‐Correcher et al. 2026), and should be considered when assessing feeding preferences of herbivores active throughout the growing season. For example, in our study system it would be interesting to assess whether pika preferences differ between early and late season, when pikas are respectively feeding or actively collecting plants for overwinter storage.

In the cafeteria trials, caterpillars showed a preference to forage on male *S. arctica* but pikas did not show sex‐biased preferences. Forage selection in pikas is affected by a complex suite of factors that are species‐specific (Morrison and Hik 2008) and is affected by interactions with other herbivores. For instance, pikas are known to forage more in patches previously used by caterpillars (Barrio, Hik, et al. 2013), and for some plant species, they actively select leaves damaged by invertebrates over non‐damaged leaves (Peck 2012). Preference by mammalian herbivores for plants previously attacked by invertebrate herbivores has also been demonstrated for the vole *M. agrestis* (Danell et al. 1985). If pikas are selecting plants that are damaged by caterpillars earlier in the season, their selection under natural settings could indeed be biased to males damaged by caterpillars earlier in the season, and the combined effect of subsequent herbivores could further reinforce the female‐biased sex ratios in the Arctic willow. However, this possibility remains speculative and should be explored in future studies assessing the preferences of pikas using a factorial design crossing plant sex and previous herbivory by invertebrates.

It is important to note that our study has some limitations, including a relatively small sample size and that it was conducted at a single location, as well as some inherent constraints of our experimental set ups. Future studies should increase sample sizes and the geographical representation, and include other herbivore species, to enhance the generalizability of our results. In our study we used cafeteria trials to assess herbivore preferences that have been previously tested and successfully applied to the two study species (caterpillars: Barrio et al. 2015; pikas: Morrison et al. 2004; Koh and Hik 2007; Hudson et al. 2008; Morrison and Hik 2008). One caveat is that the trials differed between the two herbivores: while the only food caterpillars had available during the trials was the plant samples, pikas had still other food choices available. Although this could have made pikas less selective, similar experimental set ups where pikas have access to other food sources have been used to effectively demonstrate feeding preferences for certain plant species (Morrison et al. 2004), leaf traits (Hudson et al. 2008), and plant quality (Koh and Hik 2007; Morrison and Hik 2008). Therefore, we believe the lack of sex‐biased preferences in pikas is not an artifact of our study design. Future studies should also include assessments of feeding preferences for caterpillars mimicking foraging choices in the field, where caterpillars have access to a broader range of species than in our cafeterias.

Despite these limitations, our study suggests that feeding preferences of sequential herbivores could reinforce the female‐biased sex ratio in an alpine willow, but of course further research is needed. More broadly, our study contributes to the growing body of evidence that sex‐specific responses should be considered in ecological studies when dioecious species are involved (Gissi et al. 2024). Recent advances in the field have shown that plant sex can influence belowground microbial communities (Guo et al. 2023), which can lead to facilitative belowground interactions (Xia et al. 2024). In Arctic dioecious species like *S. arctica* , males and females exhibit distinct physiological traits and habitat preferences that are linked to differences in nutrient uptake, water relations, and carbon allocation (Dawson and Bliss 1989; Jones et al. 1999). These sex‐specific traits likely condition rhizosphere microbial assemblages differently by modulating root exudation quantity and quality as well as nutrient demands, thereby influencing soil microbial biomass and community composition with downstream effects on nutrient cycling and plant–soil feedbacks (Guyonnet et al. 2018; Tejnecký et al. 2025). This mechanistic linkage reinforces the concept that plant sex in arctic tundra systems is an important driver shaping belowground biotic interactions and ecosystem nutrient dynamics. Further, the ecophysiological differences between male and female plants can lead to contrasting responses to environmental changes, including different herbivore pressures (Jones et al. 1999). These differences have ramifications to climate change adaptations of plants and the conservation and restoration of certain ecosystems, like riparian systems (Scheuerell and LeRoy 2023). In highly seasonal environments, the cumulative effects of different herbivores on plant population dynamics should be examined in more detail, particularly if the relative balance of caterpillar and pika herbivory changes in the future.

## Author Contributions

**I. C. Barrio:** conceptualization (equal), data curation (equal), formal analysis (equal), investigation (equal), methodology (equal), visualization (equal), writing – original draft (equal). **C. G. Bueno:** conceptualization (equal), investigation (equal), methodology (equal), writing – review and editing (equal). **D. S. Hik:** conceptualization (equal), funding acquisition (equal), resources (equal), writing – review and editing (equal).

## Funding

This work was supported by an AXA Research Fund postdoctoral fellowship and an honorary Killam fellowship from the University of Alberta to C.G.B.; Natural Sciences and Engineering Research Council of Canada to D.S.H.; I.C.B. was supported by the Consejería de Educación, Ciencia y Cultura (JCCM, Spain) and the European Social Fund, and the Percy Sladen Memorial fund.

## Conflicts of Interest

The authors declare no conflicts of interest.

## Supporting information


**Figure S1:** Visual assessment of model residuals allowed confirming that modeling assumptions were met, for both the caterpillar (A) and the pika (B) models.

## Data Availability

The dataset and codes are publicly available in Dryad (https://doi.org/10.5061/dryad.bg79cnprr), as well as in a public GitHub repository: https://github.com/icbarrio/sex‐biased_herbivory.git.
