# Supplementary material for: Can Herbivore Feeding Preferences Reinforce the Female‐Biased Sex Ratio in an Alpine Willow?
Source: Ecol Evol. 2026 Jun 8;16(6):e73816. doi: 10.1002/ece3.73816 (PMC13244070; doi:10.1002/ece3.73816)
Supplement: Supplementary file 1 — Figure S1: Visual assessment of model residuals allowed confirming that modeling assumptions were met, for both the caterpillar (A) and the pika (B) models. [file ECE3-16-e73816-s001.docx]

**Supplementary materials**

**Figure S1.** Visual assessment of model residuals allowed confirming that modelling assumptions were met, for both the caterpillar (A) and the pika (B) models.

| **A. Caterpillar models** |
| --- |
| **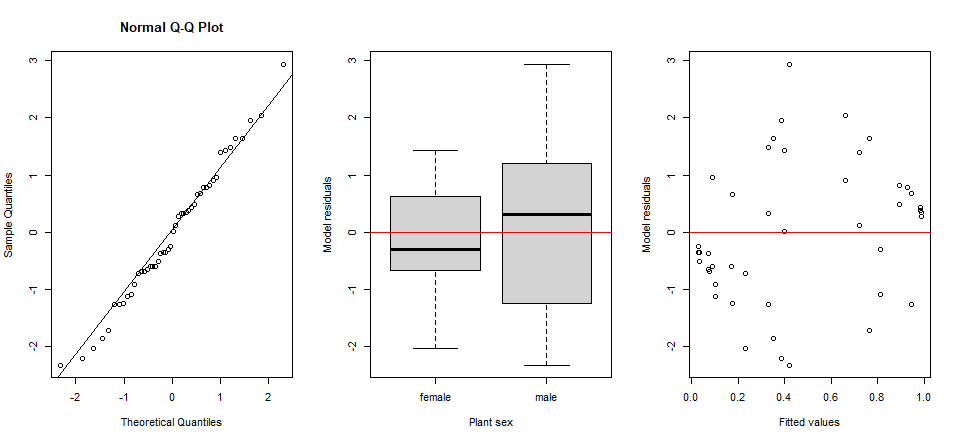** |
| **B. Pika models** |
| **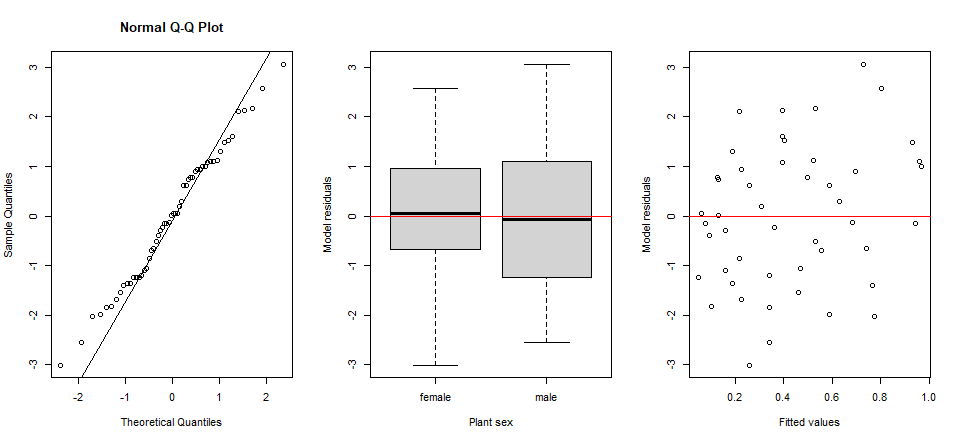** |
